# Amplitude gating for a coached breathing approach in respiratory gated 10 MV flattening filter‐free VMAT delivery

**DOI:** 10.1120/jacmp.v16i4.5350

**Published:** 2015-07-08

**Authors:** Francis Viel, Richard Lee, Ermias Gete, Cheryl Duzenli

**Affiliations:** ^1^ Department of Physics and Astronomy University of British Columbia Vancouver BC; ^2^ Department of Medical Physics British Columbia Cancer Agency– Vancouver Centre Vancouver BC Canada

**Keywords:** quality assurance, respiratory gating, respiratory coaching, volumetric‐modulated arc therapy, flattening filter free

## Abstract

The purpose of this study was to investigate amplitude gating combined with a coached breathing strategy for 10 MV flattening filter‐free (FFF) volumetric‐modulated arc therapy (VMAT) on the Varian TrueBeam linac. Ten patient plans for VMAT SABR liver were created using the Eclipse treatment planning system (TPS). The verification plans were then transferred to a CT‐scanned Quasar phantom and delivered on a TrueBeam linac using a 10 MV FFF beam and Varian's real‐time position management (RPM) system for respiratory gating based on breathing amplitude. Breathing traces were acquired from ten patients using two kinds of breathing patterns: free breathing and an interrupted (~5 s pause) end of exhale coached breathing pattern. Ion chamber and Gafchromic film measurements were acquired for a gated delivery while the phantom moved under the described breathing patterns, as well as for a nongated stationary phantom delivery. The gate window was set to obtain a range of residual target motion from 2–5 mm. All gated deliveries on a moving phantom have been shown to be dosimetrically equivalent to the nongated deliveries on a static phantom, with differences in point dose measurements under 1% and average gamma 2%/2 mm agreement above 98.7%. Comparison with the treatment planning system also resulted in good agreement, with differences in point‐dose measurements under 2.5% and average gamma 3%/3 mm agreement of 97%. The use of a coached breathing pattern significantly increases the duty cycle, compared with free breathing, and allows for shorter treatment times. Patients' free‐breathing patterns contain considerable variability and, although dosimetric results for gated delivery may be acceptable, it is difficult to achieve efficient treatment delivery. A coached breathing pattern combined with a 5 mm amplitude gate, resulted in both high‐quality dose distributions and overall shortest gated beam delivery times.

PACS number: 87.55.Qr

## I. INTRODUCTION

It has been well established that respiration can cause considerable liver motion of up to four centimeters in the superior–inferior direction and it is desirable to account for this motion for the purpose of increasing precision in radiation therapy.^1^, ^2^, ^3^, ^4^ Different motion management techniques offer the possibility of reducing the planned target volume (PTV) and potentially decreasing toxicity to normal tissue. The use of respiratory gating, for example, limits the beam delivery to a specific portion of the respiratory cycle. Doing so necessitates many interruptions of the beam, which results in significantly increased treatment time. To compensate for this limitation, the idea of coupling respiratory gating with a fast delivery technique, such as volumetric‐modulated arc therapy (VMAT), has increasingly been adopted as the treatment of choice for moving tumors in the liver and the pancreas.^5^, ^6^, ^7^, ^8^ In addition, the new advancements in flattening filter‐free mode (FFF) for modulated treatments allow for an increased dose rate, thus significantly reducing the treatment time for treatments delivering high doses per fraction, such as stereotactic ablative radiation therapy (SABR).^(9)^ The combination of reduced margins and gated VMAT could permit treatment of a select group of hepatocellular carcinoma (HCC) patients who were previously ineligible for radiation therapy due to larger lesions.^(10)^

In previous studies, Qian et al.^(7)^ and Nicolini et al.^(8)^ showed the feasibility of gated VMAT and the dosimetric accuracy under different gating periods on a static phantom. Li et al.^(5)^ and Choi et al.^(6)^ investigated the geometric accuracy of gated VMAT using kV imaging and a dynamic phantom. Respiratory motion during intensity modulation, such as IMRT or VMAT, presents an additional challenge over 3D conformal therapy as dose gradients are no longer limited to the edges of the field and vary with time. In these cases, tumor and organs at risk are likely to move through high‐dose gradients even within the gate. For this reason it is important to also consider the residual motion when performing dosimetric measurements.

Gated IMRT has been verified with film measurements on dynamic phantoms undergoing sinusoidal motion.^11^, ^12^ Oliver et al.^(11)^ developed a Monte Carlo program to simulate segmental IMRT delivery to a moving phantom, while Lee et al.^(12)^ tested gated IMRT with different gate window widths using film measurements on a phantom undergoing sinusoidal motion. The dosimetric impact due to the interplay effect (defined as the simultaneous motion of the target and the dynamic multileaf collimator) has been evaluated to be of lesser significance than the variability in the respiratory motion during gated VMAT.^(13)^ These investigations provided an assessment of the effect of residual motion within the gate which is essential for patient‐specific quality assurance (QA) prior to treatment.

Riley et al.^(13)^ noted that greater irregularity in patient breathing pattern was correlated with poorer quality dose distributions. This could be a more significant problem with phase gating as opposed to amplitude gating. To our knowledge, no one has assessed the dosimetric accuracy and timing of amplitude gated VMAT for a coached breathing strategy versus free breathing using actual patient breathing traces to simulate the residual motion with a moving phantom. The goal of this study was to compare various gate windows for both free breathing and coached breathing in terms of the dose to a moving volume and treatment delivery time. This study was an essential component in the clinical implementation of gated FFF VMAT SABR treatments to otherwise untreatable HCC liver tumors at the Vancouver Centre of the British Columbia Cancer Agency.^14^, ^15^

## II. MATERIALS AND METHODS

**Figure 1:**
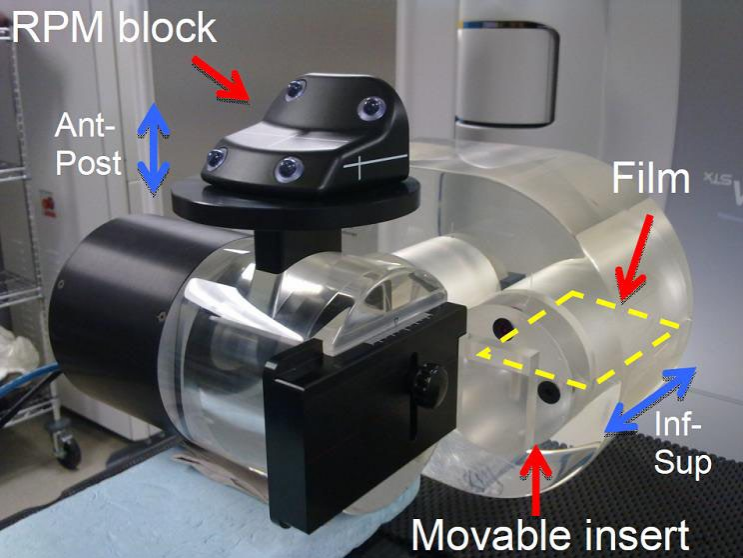
Picture of the Quasar phantom. The insert containing the film moves in the inferior–superior direction and the top platform moves in the anterior–posterior direction for supporting the RPM block.

The investigation in this work was carried out using a Varian TrueBeam linac (Varian Medical Systems, Palo Alto, CA) and a Quasar (Modus Medical Devices Inc., London, ON, Canada) phantom. The Quasar respiratory motion phantom is a motorized phantom that can reproduce the motion from a recorded patient breathing trace. The phantom has interchangeable inserts that can move in the inferior–superior direction according to the input breathing trace, as well as the capability of performing more complex nonlinear motion. Various inserts are available to hold a piece of film approximately $7\times 11\;{\text{cm}}^{2}$ or an ion chamber. The respiratory motion is also reproduced in the anterior–posterior direction by a platform to simulate the chest motion, as shown in Fig. 1. The relationship between the motion of the insert and the platform is adjustable, but was kept at a fixed position with a 1:1 gearing ratio.

### A. Treatment planning

Ten realistic VMAT SABR liver patient treatment plans were created using the Eclipse treatment planning system (TPS) (Varian Medical Systems) as part of a previous planning study.^(10)^ The Eclipse anisotropic analytical algorithm (AAA) (version 10.0.28) was used for dose calculation with a 10 MV FFF beam using one full or partial VMAT arc. Treatment planning parameters are shown in Table 1. Verification plans were then created on the Quasar respiratory motion phantom on the 50% (end of exhale) phase of a 4D CT (4D computed tomography) scan of the phantom and the isocenter of each plan was placed at the center of the insert.

**Table 1:** Summary of the plans' characteristics. All plans had a prescribed dose of 300 cGy and maximum gantry speed of $4.8^{{}^{\circ}}/s$

		*Jaw Setting*				
*Plan #*	*PTV Size* (${\text{cm}}^{3}$)	*X (cm)*	*Y (cm)*	*MU (MU)*	*Arc Angle (CCW) (°)*	*Approximate Average Dose Rate (MU/min)*
1	60	6.7	6.7	740	180→315	950
2	378	13.3	11.0	1121	90→180	1200
3	2388	16.5	19.5	957	90→181	1000
4	154	8.8	8.6	757	180→180	600
5	280	11.7	12.3	1183	180→180	970
6	871	15.7	15.7	591	100→181	600
7	186	10.2	10.4	925	180→252	1100
8	942	16.8	14.3	969	100→181	1000
9	216	11.3	12.0	957	179→181	770
10	1401	16.5	17.9	774	180→181	700

### B. Acquisition of patient breathing traces

**Figure 2:**
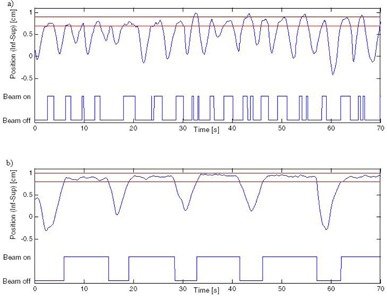
Sample breathing patterns with a 2 mm gate: a) free breathing (FB) and b) interrupted end of exhale coached breathing (BIBOP). The maximum position corresponds to exhale and the minimum to inhale.

Under research ethics board approval, ten patients provided two breathing traces each: one as a free‐breathing (FB) pattern and one as an interrupted (~5−10 s pause) end of exhale coached breathing pattern (also referred to Breathe‐In Breathe‐Out Pause, BIBOP, in this paper). The breathing traces were recorded using Varian's Real‐Time Position Management System (RPM). The coached breathing pattern results in a larger duty cycle during gating when compared to the FB pattern and can lead to shorter treatment times.^(16)^ Figure 2 shows an example of the breathing traces in the BIBOP and FB mode typical of the patient group. During recording of the BIBOP trace, the patients were asked to hold their breath for approximately 5 to 10 s at end of exhale, inhale once and repeat the 5 to 10 s hold at each exhale. A screen was mounted on the simulation couch to display the breathing pattern to the patients in real time to improve breathing reproducibility.^17^, ^18^, ^19^ A 4D CT of the patient under free‐breathing conditions was also obtained at this time.

### C. Measurements

Ion chamber and Gafchromic film measurements were performed for a gated delivery while the phantom mimicked the motion of the breathing traces of the patient. In order to achieve this, the patient's breathing trace was rescaled until the superior–inferior motion of the phantom agreed with the amplitude of motion as measured from the patient's 4D CT. The same plan was delivered to a static phantom without gating, hereafter known as static delivery conditions. This enabled a direct comparison of the effect of phantom movement and gating vs. static conditions, allowing us to isolate and separately investigate the possible machine performance issues associated with gated delivery and any residual interplay effect, excluding any confounding effects from the dose calculation engine in the TPS. In addition, the nongated static deliveries and gated deliveries were compared to the TPS to verify that the treatment was delivered as planned. Gated deliveries were executed using amplitude gating and the gate window was set to obtain a range of residual target motion from 2–5 mm at end of exhale.

In brief, for each patient plan, Gafchromic film and ion chamber measurements were performed under different gating parameters and breathing traces for a total of six combinations: a nongated delivery with no motion, a nongated delivery under FB motion, and four gated deliveries with a 2 mm and a 5 mm window under FB and BIBOP motion.

### D. Gafchromic film

#### D.1 Film to film comparison

Film measurements in the coronal plane were obtained using Gafchromic EBT3 film (Ashland, Covington, KY) placed inside an in‐house built homogeneous insert for the Quasar phantom. The films were scanned using an Epson Expression 10000 XL flatbed scanner (US Epson, Long Beach, CA). RGB images were collected in reflective mode with 16 bits per color channel and a spatial resolution of 150 dpi corresponding to a pixel size of $0.17\times 0.17\;{\text{mm}}^{2}$. Film calibration and analysis were performed using the protocol developed by Bouchard et al.,^(20)^ which uses the red and blue channels. The calibration curve was created based on pieces of film of $2\times 3\;{\text{cm}}^{2}$ irradiated to a known dose with a $10\times 10\;{\text{cm}}^{2}$ field in a Solid Water phantom at 10 cm depth. Gamma analysis was used to compare the film measurements of the gated deliveries with the static nongated deliveries. The parameters were chosen to be 3%/3 mm, 2%/2 mm, and 1%/1 mm for all the pixels in a $6\times 10\;{\text{cm}}^{2}$ region of interest.

#### D.2 Film to TPS comparison

Partway through the study, the FilmQA Pro software (Ashland) became available and was used for comparison of the film measurements with the TPS. Film measurements were repeated under static conditions and using the coached breathing trace with a 5 mm window for gated deliveries. RGB images were collected in transmission mode with 16 bits per color channel and a spatial resolution of 72 dpi corresponding to a pixel size of $0.35\times 0.35\;{\text{mm}}^{2}$. The film measurements were compared with the TPS‐calculated dose maps using gamma analysis at 3%/3 mm and 2%/2 mm for a rectangular region of interest contained within the main body of the phantom and fitting around the 80% of maximum dose isolines, resulting in a region of interest ranging from $6\times 7\;{\text{cm}}^{2}$ to $6\times 9\;{\text{cm}}^{2}$.

### E. Ion chamber

Ion chamber measurements were performed using a Farmer‐type ion chamber with an active volume of 0.6 cc (PTW‐Freiburg, Germany). The ion chamber was placed in a cylindrical insert within the Quasar phantom. The point of measurement was 1.5 cm to the left of the center of the insert and was kept within the high‐dose region in all cases. After each set of measurements, ion chamber cross‐calibration was done by delivering a known dose to a static chamber inside the Quasar phantom with an open field.

### F. Treatment time

The treatment times were measured from the moment that the “Beam On” button was pressed up to the complete delivery of the requisite MUs. They do not include patient setup and coaching or the time necessary for the RPM system to “learn” the breathing pattern. Further, the beam‐on time was also recorded from the TrueBeam treatment application for six of the ten plans, thus allowing for the calculation of the duty cycle defined as the ratio of the beam‐on time over the treatment time.

## III. RESULTS

### A. Film

**Figure 3:**
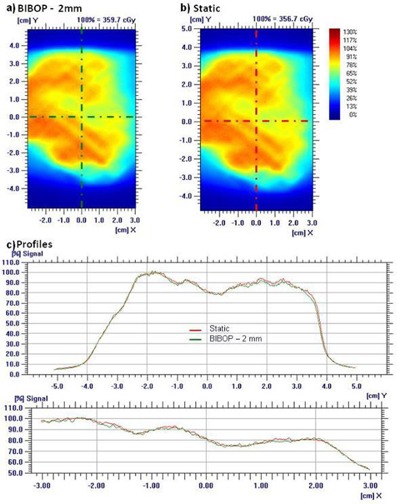
Typical example of a film measurement. The dose maps measured for Plan 4 with prescribed dose of 300 cGy with coached breathing with 2 mm gate width (a) and static (b) conditions are shown with their X and Y profiles (c) on the central axis.

Figure 3 shows a typical film to film comparison for Plan 4 with a prescribed dose of 300 cGy delivered with coached breathing and 2 mm gate width on a moving phantom, compared to static conditions. There was no apparent shift or blurring of the dose and no major differences can be seen between the static and the gated deliveries under any of our test conditions. The differences in relative doses do not exceed 5%.

**Figure 4:**
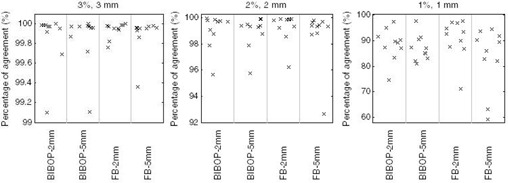
Gamma analysis results of the gated deliveries compared to the static delivery. Note that the three plots are displayed using different scale.

Gamma analysis 3%/3 mm, 2%/2 mm, and 1%/1 mm were used to compare the measured dose distributions of gated deliveries with the static delivery. Table 2 and Fig. 4 present the gamma percentage of agreement averaged over all the ten plans, comparing gated versus static deliveries. Gamma analysis 3%/3 mm exhibits an average percentage of agreement over 99.9%, while 2%/2 mm yields greater than 98.7% agreement. It can be seen from Fig. 4 that there was no significant difference in the percentage of agreement between the different gating conditions. Furthermore, the nongated deliveries under a free‐breathing motion resulted in a lower agreement: 93% and 81% for gamma 3%/3 mm and 2%/2 mm, respectively, indicating that there is dosimetric improvement by performing gated delivery.

**Table 2:** Gamma analysis results of the gated deliveries compared to the static delivery. Average of the percentage of agreement over all plans with range in brackets

*Breathing Pattern*	*Gate Width (mm)*	3%/3 mm *(%)*		2%/2 mm *(%)*		1%/1 mm *(%)*	
BIBOP	2	99.9±0.1	(99.1–100.0)	99.0±0.4	(95.7–100.0)	89±2	(74.6–97.3)
BIBOP	5	99.9±0.1	(99.1–100.0)	98.9±0.4	(95.8–99.9)	87±2	(80.9–97.8)
FB	2	99.9±0.1	(99.8–100.0)	99.3±0.4	(96.2–99.9)	91±2	(71.3–97.8)
FB	5	99.9±0.1	(99.4–100.0)	98.7±0.7	(92.7–99.8)	83±4	(59.3–94.5)
FB	Nongated	93±2	(82.6–99.1)	81±3	(66.9–94.6)	56±3	(44.1–72.1)

**Figure 5:**
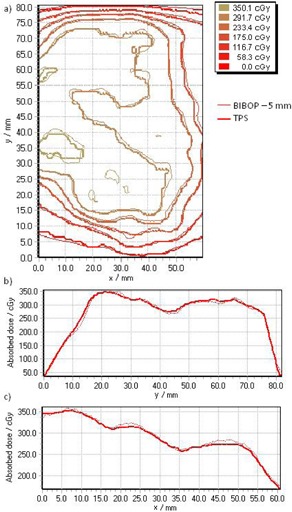
Typical example of a film comparision with the treatment planning system. The dose isolines (a) measured and calculated by the TPS for Plan 4 are shown with their Y (b) and X profiles (c) on the central axis.

**Figure 6:**
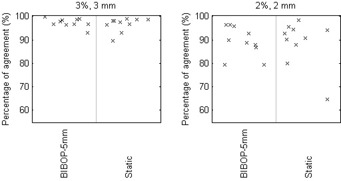
Gamma analysis results of the gated and static deliveries compared to the treatment planning system.

A comparison between the BIBOP–5 mm gated delivery and the TPS was also performed. Figure 5 shows the comparison between the measured and TPS‐calculated dose distributions for Plan 4. Gamma analysis gave agreements of 97% 3%/3 mm and 89% 2%/2 mm, on average (Table 3 and Fig. 6). Further analysis using the remaining combinations of breathing patterns and gate widths with the TPS was not performed since they were all in good agreement with the static case.

**Table 3:** Gamma analysis results of the gated and static deliveries compared to the treatment planning system. Average of the percentage of agreement over all plans with range in brackets

*Breathing Pattern*	*Gate Width (mm)*	3%/3 mm *(%)*	2%/2 mm *(%)*
BIBOP	5	97±1 (93.0–99.8)	89±2 (79.3–96.4)
Static	–	97±1 (89.5–98.9)	89±3 (64.6–98.5)

### B. Ion chamber

**Figure 7:**
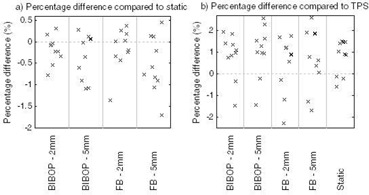
Percentage difference in point dose from ion chamber measurements.

Point‐dose measurements of gated deliveries were compared to the point doses calculated with the TPS and to the static point dose measurements. The results are summarized in Table 4 and shown in Fig. 7. The agreement between ion chamber measurements for the gated and static deliveries was within 2% for all breathing pattern and gate width combinations (Fig. 7(a)). Further, the ion chamber measurements were within 2.6% of the planned dose as calculated by the TPS in all cases (Fig. 7(b)) and within 1%, on average.

**Table 4:** Percentage difference in point dose from ion chamber measurements

*Breathing Pattern*	*Gate Width (mm)*	*Ion Chamber Gated Compared to Static (%)*	*Ion Chamber Gated Compared to TPS (%)*
BIBOP	2	−0.2±0.1 (−0.8–0.3)	0.8±0.3 (−1.5–1.9)
BIBOP	5	−0.4±0.2 (−1.1–0.3)	1.0±0.4 (−1.5–2.6)
FB	2	−0.2±0.2 (−1.4–0.4)	0.4±0.4 (−2.3–1.7)
FB	5	−0.5±0.2 (−1.7–0.5)	0.7±0.4 (−1.7–2.6)
Static	–	–	0.8±0.3 (−0.6–1.5)

### C. Treatment time

**Figure 8:**
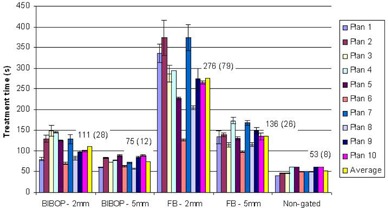
Average treatment time per plan. The overall average for all plans is displayed in yellow with its value in seconds on the graph (the SD is shown in brackets).

When compared to static deliveries, gated deliveries can significantly increase the treatment time due to the interruptions of the beam. The number and duration of the interruptions are highly dependent on the breathing trace and the gating parameters. Figure 8 shows the average treatment time for different breathing patterns and gate windows.

As one would expect, treatment time increases with decreasing gate windows, simply due to the beam being on for a shorter portion of the breathing cycle. With the free‐breathing pattern, the average delivery time was approximately twice as long when the gate width was reduced from 5 mm to 2 mm. Using the coached breathing pattern with a 5 s breath‐hold at end of exhale can reduce the treatment time by more than a factor of two compared to free breathing with a 2 mm window. It can be seen from Fig. 8 that the coached breathing pattern can also reduce the variability in treatment time that resulted from the high variability of the free‐breathing patterns. The free‐breathing patterns not only differ from patient to patient, but they can also vary for the same patient when comparing different treatment sessions. This is evident in Fig. 8, which exhibits greater treatment time variability per patient for FB–2 mm.

**Figure 9:**
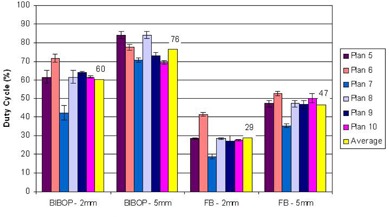
Average duty cycle (beam‐on time over treatment time) per plan. The overall average for all plans is displayed in yellow with its value in percent on the graph.

Figure 9 shows the average duty cycle per plan for different breathing patterns and gate windows. The duty cycle is higher for coached breathing than free breathing, and a gate width of 5 mm produced the highest duty cycle overall.

## IV. DISCUSSION

This study is the first to investigate the efficacy and the dosimetric accuracy of using coached breathing, with a short pause at the end of exhale (BIBOP), for VMAT FFF SABR plans delivered with the amplitude gating technique. Our dosimetric results comparing gated to nongated delivery indicate >99% 3%/3 mm gamma pass rate for all cases. No trend is seen with PTV volume, for volumes ranging from 60 cc to 2388 cc. Our study was based on actual patient breathing traces, while most previous studies^7^, ^11^, ^13^ used a sinusoidal breathing pattern, which may mask problems associated with realistic breathing patterns. These previous studies also used a phase gating approach. Riley et al.^(13)^ found three out of ten VMAT SABR cases failed the 3%/3 mm gamma test when using realistic patient breathing traces. Qian et al.^(7)^ observed gamma passing rates >95% using only sinusoidal breathing traces for VMAT plans delivered on a TrueBeam linac. Similar dosimetric results were reported by Oliver et al.^(11)^ for IMRT plans delivered on a Varian 2100 C/D. One observation of note by Riley et al.^(13)^ was that irregular breathing could be responsible for poor dosimetric results seen in his phase gated study. This is a recognized limitation of phase gating, which is aggravated with irregular breathing. Amplitude gating mitigates this effect, as the residual motion is limited to the width of the gate as long as the relationship between the internal target and external surrogate remain constant. This is consistent with our results which indicate that amplitude gating produces a better dosimetric outcome regardless of the irregularity of the breathing pattern.

Amplitude gating may introduce longer delivery times which could be a more important consideration in the context of SABR type fractional doses and for conventional dose rates. The introduction of coached breathing, with a short pause at the end of exhale (BIBOP), restores the short delivery times promised with RapidArc and FFF beams. Our results indicate that a delivery time of ~75s for a 5 mm gate is achievable with coached breathing, which compares well with 53 s for a nongated delivery. In addition, the breathing pause will effectively eliminate any potential interplay effects since the target is effectively stationary for the majority of the field's duration.

Fast delivery is not only for convenience but also has potential dosimetric implications. A prolonged treatment time increases the likelihood of patient motion during treatment. A relatively short treatment time also reduces the amount of drift in the overall breathing pattern. In addition to shorter treatment times, BIBOP also contributes to a reduction in the amount of residual motion within the gate window. Regardless of the width of the window, the short breath‐hold during BIBOP keeps the target nearly stationary. Therefore, while free breathing with a 2 mm window physically limits the residual motion to 2 mm, the BIBOP–5 mm can be delivered faster, while maintaining an acceptable residual motion.

It is interesting to note that the beam‐on time for a gated delivery is often longer than the delivery time of a nongated delivery. This may be due to the extra time it takes for the dose rate to ramp up every time the beam comes on after an interruption. This ramp‐up takes around 0.6 s as observed from the trajectory log files. The added effect is then larger when there are many interruptions and when the individual beam‐on windows are short, as is the case for FB–2 mm where the beam‐on time can take up to 25% longer than the corresponding nongated delivery. On the other hand, using BIBOP–5 mm resulted in beam‐on times identical to the nongated treatment times.

Continuing development of the QA procedure for amplitude gated FFF VMAT is ongoing as we have identified some limitations of the technique during the course of this study. The insert of the Quasar phantom can extend outside of the main body of the phantom and is too small for very large lesions. In some of the cases tested, the lesion is sufficiently large that the film did not cover the entire high‐dose region. Furthermore, the superior part of the film (~1 cm) inside the movable insert can extend past the main body of the phantom during exhale. This can lead to inaccurate calculation from the TPS in this region. In order to address this limitation, a programmable moving platform that is capable of supporting phantoms up to $30\times 30\times 30\;{\text{cm}}^{3}$ is currently under development in‐house. In addition, a newer version of the Quasar phantom that addresses this problem is now available (Quasar Cylindrical Respiratory Motion Phantom from Modus Medical Devices Inc.).

While the use of reflective mode for the film scanning under Bouchard's protocol is not in common use, reflective mode has been examined in previous papers^20^, ^21^ to produce comparable results to transmission mode.^(22)^ While one study^(23)^ has suggested a dependence of the dose in an ROI to the pixel intensity of any surrounding film, Mendez et al.^(21)^ has not corroborated the finding and believed it may be a scanner‐specific effect. We have also not observed any significant difference in the accuracy of the two modes as long as the appropriate procedures were followed. Other shortcomings were noted in relation to the use of Bouchard's software for film analysis. Since it was initially written for use with the Gafchromic EBT film, only the red channel is used for converting optical densities to dose and its signal saturates at doses higher than 800 cGy.^(20)^ Therefore, the software is not usable for higher doses as with hypofractionated treatments. This constraint required a reduction of the dose to 300 cGy from the original prescription. The sole result of the change in dose was a rescaling of the dose rate; no alteration to the MLC leaf positions, gantry speeds or treatment time was observed. There was no such limitation present in Ashland's FilmQA Pro due to its use of triple channel dosimetry and its “one scan” protocol.^(22)^ Bouchard's protocol also is capable of a higher degree of accuracy (~1% compared to ~2% from FilmQA Pro), but the protocol is more labor intensive, requiring the average of multiple film scans as well as scanner inhomogeneity corrections. We have also observed that periodic reacquisition of the scanner inhomogeneity was necessary, as well as recalibration of the OD‐dose curve three to four times a year, even for the same batch of film. Due to this combination of limitations, we are currently in the process of transitioning to the use of FilmQA Pro for all further film analyses at our institution.

## V. CONCLUSIONS

The results of this study indicate that coached breathing with end of exhalation breath‐holds and a 5 mm amplitude gate window produce clinically acceptable dose distributions while maintaining efficient treatment deliveries for gated VMAT SABR with 10 MV FFF beams. From November 2013 to August 2014, seven liver SABR patients have now been treated at our institution, using amplitude gated VMAT with 10 MV FFF and a BIBOP coached breathing pattern with a window of 5 mm. Film dosimetry comparing gated to nongated delivery for these patient cases resulted in gamma 3%/3 mm agreement from 98.1% to 100% and 2%/2 mm agreement from 97.6% to 99.7%.

## ACKNOWLEDGMENTS

The authors would like to thank Hugo Bouchard for the use of his film analysis software and Ashland for granting a temporary license of the FilmQA Pro software. Acknowledgements are due also to the machine shop at the BCCA — Vancouver Centre for designing and producing a personalized insert for the Quasar phantom and to the radiation therapists who collected the breathing traces. This work was partly supported by the Varian Research Collaborations Program.
